# Review of 175 Cases of Tuberculosis Infections Affecting the Urogenital System

**DOI:** 10.5152/tud.2022.22148

**Published:** 2022-11-01

**Authors:** Nuno Dias, Teresa Pina-Vaz, Pedro Abreu-Mendes, Tiago Azeredo-Costa, Pedro Rodrigues-Pereira, Carlos Silva, Francisco Botelho

**Affiliations:** 1 Department of Urology, São João Hospital Center, Porto, Portugal; 2 Department of Surgery and Physiology Porto University Faculty of Medicine, Porto, Portugal; 3 Department of Clinical Pathology, São João Hospital Center, Porto, Portugal; 4 Department of Pathology, São João Hospital Center, Porto, Portugal; 5 Life and Health Sciences Investigation Institute, Minho University Faculty of Medicine, Braga, Portugal

**Keywords:** Urinary tract infections, tuberculosis, urogenital, *Mycobacterium tuberculosis*

## Abstract

**Objective:**

Urogenital tuberculosis and disseminated tuberculosis affecting urogenital system are more frequent in developing countries but are often overlooked in developed ones. We aimed to compare clinical characteristics and outcomes of urogenital tuberculosis and disseminated tuberculosis affecting the urogenital system.

**Material and methods:**

We retrospectively reviewed data from patients with tuberculosis in the urogenital system, diagnosed in a tertiary center in a European country, from 2008 to 2018. Cases were divided into urogenital tuberculosis and disseminated tuberculosis affecting the urogenital system and compared.

**Results:**

We included 172 patients, 31 with urogenital tuberculosis and 141 with disseminated tuberculosis affecting urogenital system. Patients with disseminated tuberculosis affecting the urogenital system were younger (median 45 vs. 64 years, *P* = .001), more likely to be male (80 vs. 55%, *P* = .005), or having risk factors for the disease (84 vs. 23%, *P* = .005) than patients with urogenital tuberculosis. Patients with urogenital tuberculosis presented most commonly with symptoms related to the urinary tract, with 52% complaining of lower urinary tract symptoms attributed to urinary tract infections and 48% of dysuria, while patients with disseminated tuberculosis affecting the urogenital system presented mainly with systemic symptoms, with 89% complaining of malaise, 62% of fever, and 57% of anorexia. Patients with urogenital tuberculosis were more likely to need urological surgery as part of their treatment (71 vs. 5%, *P* < .001) and patients with disseminated tuberculosis affecting the urogenital system were more likely to die due to tuberculosis (10 vs. 21%, *P* < .001).

**Conclusion:**

Tuberculosis of the urogenital system can have multiple clinical presentations, and a simple diagnostic algorithm does not exist. In the presence of urogenital tuberculosis in injected drug users, immunosuppressed individuals, or patients with systemic symptoms, we should think of disseminated tuberculosis affecting the urogenital system and remember these patients less frequently need surgery but entail a worst outcome.

**Main Points**

- Patients with tuberculosis affecting urogenital systems can present with localized (urogenital tuberculosis) or systemic disease (disseminated tuberculosis affecting urogenital system).
- Patients with urogenital tuberculosis present more commonly with urinary symptoms, such as dysuria and storage symptoms, while patients with disseminated tuberculosis affecting urogenital system generally present systemic symptoms such as malaise, anorexia, and fever.
- There is no single method of diagnosis for urogenital tuberculosis with sensitivity > 85%.
- Clinicians should remember *Mycobacterium tuberculosis complex* infection as a possible diagnosis in atypical presentations or persistent symptoms after adequate treatment.
- In the presence of patients with systemic symptoms, we should be aware of the possibility of disseminated tuberculosis and remember that these patients need surgery less frequently but entail a worst disease outcome.

## Introduction

Tuberculosis (TB) is a disease caused by infection with *Mycobacterium tuberculosis (Mycobacterium tuberculosis* *)*. According to the World Health Organization, a third of the population has been infected with *MTb* but is not ill and cannot transmit the disease (latent TB).^1^ Those have a lifetime risk of 10% of developing TB, thus posing a greater threat for the immunocompromised population.

Worldwide, TB is the second most common infectious cause of death and is present mainly in developing countries. In Portugal, although TB infection incidence has decreased more than 50% between 2000 and 2015, it was still the highest incidence rate and prevalence among European countries in 2015, particularly in Lisbon and Northern region, with an incidence of 17.8/100,000 inhabitants, classifying it as an intermediate endemic disease.^2^

It is hypothesized that after inhalation or ingestion of *Mycobacterium tuberculosis complex *(*MTc*) bacilli, these multiply locally in the tissues, forming a primary granuloma, and stay contained as latent TB in most people. Five to ten percent of people with latent TB end up having lymphatic or hematologic spread of the disease, with seeding to body tissues, resulting in extrapulmonary TB, of which urogenital TB is the most common. From this point on, it may take 1-15 years until an active infection is manifested.^3,4^

Extrapulmonary TB accounts for approximately 20% of reported cases, and in patients with pulmonary TB, urogenital involvement occurs in around 15% of cases.^5^ Urogenital TB (UGTB) results from infectious inflammation of urogenital system caused by *MTb* or *Mycobacterium bovis*. It can be clinically classified as (i) kidney TB, (ii) urinary tract TB, (iii) genital TB, or (iv) disseminated tuberculosis affecting the urogenital system (DTU).^6^

There is a lack of consensus on the best approach on defining, diagnosing, and treating this disease in its various forms due to the paucity of available literature. We aimed to review the experience of a single tertiary center with TB infections affecting the urogenital system regarding their presentation, diagnosis, surgical treatments, and prognosis and analyze if those differed between pure UGTB and DTU.

## Materials and Methods

A retrospective analysis was performed by reviewing the clinical files of all patients diagnosed with TB affecting the urogenital system recorded in our institution between 2008 and 2018.

The study has received ethical approval from the local Ethics Committee (no. 434/21). The Ethical Committee waived the need for patient consent since this was a retrospective purely observational study; the best clinical practices were observed and all the data were pseudo-anonymized.

Urogenital tuberculosis was defined when at least one of the following criteria was fulfilled: (i) presence of acid-fast bacilli in urine samples by auramine stain and Kinyoun staining method, (ii) isolation of *MTc* in urine culture in BD BBL™ MGIT™ *Mycobacterium* growth index tube, (iii) urine polymerase chain reaction (PCR) positive for *MTc*, and (iv) histopathological evidence in relevant tissue specimen, usually by identifying the triad of caseating necrosis, loose aggregates of epithelioid histiocytes, and giant Langhans cells.^6^

Disseminated tuberculosis affecting urogenital system was defined as UGTB and either presence of *MTb* in (i) 2 non-adjacent organs, (ii) blood cultures, or (iii) liver or bone biopsies.^7^

Cases were identified by searching the clinical pathology database for positive urine analysis for UGTB and by searching the anatomopathology database for surgical specimens with histopathological signs of UGTB. Patients’ characteristics such as demographics, risk factors, presenting symptoms, need for surgical intervention, method of diagnosis, and treatment outcome were analyzed. Patient follow-up data, such as survival, were retrieved from the common NHS medical records. Information about medical treatment and its side effects was not possible to retrieve in most patients as the treatment was performed outside of our hospital in a Tuberculosis Treatment Unit.

Exclusion criteria included insufficient clinical data pertaining a patient due to being unavailable in our public hospital network registers, as some patients were followed in private hospitals or community centers; and previous intravesical *Bacillus Calmette-Guérin* (BCG) administration, since it may pertain to a different clinical entity.

Characteristics of patients with UGTB and DTU were compared using chi-square analysis for categorical variables and non-parametric Mann–Whitney *U*-tests for continuous variables (age). Statistical significance in this study was set as *P* < .05. All reported *P* values are two-sided. For statistical analysis, we used IBM® Statistical Package for Social Sciences v27 software.

## Results

One hundred seventy-nine cases of TB affecting the urogenital system were identified. Four patients were excluded due to insufficient data and 3 for having TB associated with intravesical BCG administration, resulting in 172 patients being included.

Thirty-one (18.0%) patients had only UGTB and 141 (82.0%) had DTU. Patient characteristics are shown in Table 1.

### Patient Demographics

Among all patients, 130 (75.6%) patients were male, 20 were diagnosed with UGTB and 110 with DTU, with male gender being more common in DTU patients (*P* = .005).

The median age at diagnosis was 47 for all patients. Patients with UGTB were older than DTU patients (64 vs. 45 years; *P* = .001).

### Risk Factors

The overall presence of risk factors was more common in the DTU group (83.4 vs. 22.6%, *P* = .005). Among the patients with DTU, 118 (83.7%) had at least 1 risk factor, with 67 (47.5%) being current or former injected drug users, 89 (63.1%) having HIV infection, and 12 (8.5%) in immunosuppressant medication other than chemotherapy or antiretrovirals. Among patients with UGTB, only 7 (23%) had risk factors, with the most common being taking immunosuppressant medication other than chemotherapy (n = 4, 13%). 

### Clinical Presentation

Clinical presentations differed between UGTB and DTU. The most common symptoms among patients with UGTB were lower urinary tract symptoms such as dysuria and urinary storage symptoms, while in DTU patients, general symptoms such as malaise, anorexia, and fever were dominant.

### Investigation and Diagnosis

Among the 141 patients with DTU, 110/141 had sterile pyuria, 115/141 had positive cultures for acid-fast bacilli, 100/127 had positive PCR for *MTc*, and 3/4 had histopathology with typical findings of TB.

Among the 31 patients with UGTB, 31 had a urinalysis and urine culture with 28 (90%) having sterile pyuria, 25 performed mycobacterilogical urine cultures with 19 (76%) being positive for acid-fast bacilli, and in 19 cases a PCR for MTc was done with 16 (84%) having positive results. Seventeen of 20 had histopathology with typical findings of TB.

Resistance to antibiotics was rare among both groups, as seen in Table 2, but it was more common in the UGTB, particularly to isoniazid and streptomycin (22% resistance for each). The percentages of multiple resistance (**≥**2 resistances) to antibiotics were not statistically different (16.7 vs. 3.7%, *P* = 0.06) between groups.

### Surgical Intervention

For 71.0% of patients in the UGTB group, at least 1 urological surgery was needed during treatment. Twelve patients (35.3%) were submitted to nephrectomy due to kidney destruction with persistent symptoms, 4 (12.9%) to orchiectomy due to testicular mass, 1 (3.2%) of them with scrotal fistula, 3 (8.8%) to cystectomy, 1 to bladder augmentation (2.9%) due to fibrosis with symptoms not controllable with medical therapy, 2 (5.9%) to ureteral stenting due to obstruction, 2 (5.9%) to bladder resection for finding of unspecific lesions, 1 (2.9%) to prostate resection due to persistent symptoms, and 1 (2.9%) to diagnostic ureteroscopy for ureteral thickening.

Among patients with DTU, from the 141 patients, only 7 (5%) needed a total of 10 surgeries. Three (2.1%) patients needed cystectomy due to fibrosis associated with symptoms not controllable with medical therapy, 2 (1.4%) needed ureteral stenting due to obstruction, 2 (1.4%) needed nephrectomies due to kidney destruction with persistent symptoms, 1 (0.7%) to ureteroscopy and then ureteral reimplantation due to distal ureter stenosis, and 1 (0.7%) was submitted to exploration laparoscopy for further workup.

### Survival Outcomes

Three months after diagnosis, 8.8% of patients with UGTB and 21.3% with DTU were deceased due to TB disease progression, with a statistically meaningful difference (*P* < .001). No patients were deceased during this timeline due to other causes.

### Clinical Data Based on Affected Organs

Among all patients, the organ most affected with TB was the kidney (n = 159, 92.4%), followed by the ureter (n = 14, 8.1%) and the bladder (n = 7, 4.1%). Genital TB was rare, with only 4 (2.3%) patients presenting with testicular or epididymal TB and 3 (1.7%) with prostate TB.

Patients with UGTB were more likely to disease of the ureter (74.1 vs. 3.5%, *P* < .001), the bladder (16.1 vs. 1.4%, *P* = .003), or the testicle/epididymis (12.9 vs. 0%, *P* < .001) and less likely to present with only kidney TB (74.2 vs. 96.5%, *P* < .001) than patients with DTU.

Two patients with UGTB presented with bilateral disease, 1 with bilateral grade 4 kidney TB, and 1 with grade 4 bladder TB and bilateral ureteral stenosis. One patient with DTU had bilateral ureteral stenosis with grade 2 kidney TB.

Further patient data according to affected organs is presented in Supplementary Table 1.

## Discussion

We described our centers’ experience with UGTB, from cases diagnosed between 2008 and 2018. We report one of the biggest series of UGTB in a European developed country setting and in a more recent cohort than most available studies. 

Patients with DTU were younger, with a median of 45 years, which is in accordance with current literature.^8,9^ Patients with UGTB had a median age of 64, with previous studies being heterogeneous, reporting median ages between 39 and 62 years.^3,10-15^

We report a male prevalence in both the UGTB and DTU population. The even higher proportion of males in DTU (80%) may be related to risk factors for the disease being more prevalent in males, and the findings were in line with those previously reported by other authors reporting a large majority of males between patients diagnosed with both UGTB and DTU.^5,10,13^ Interestingly, the presence of known risk factors for mycobacterial infection (injected drug usage, HIV infection, active chemotherapy, or other immunosuppressant medication) was common in DTU but had a low prevalence among patients with UGTB.^3^ Previous reports are rare, have patients more than 15 years, and are mainly from non-developed countries where HIV infection is more prevalent.^3^ A report from a US city in the 90s pointed to an incidence of HIV infection in patients with UGTB as high as 46%.^15^

We can see that there are not any specific symptoms related to UGTB or DTU. The clinical presentations on UGTB were mainly mild and heterogeneous, depending on which urogenital organ was most affected by the infection. The symptoms of DTU were mainly general symptoms such as malaise, anorexia, and fever, present in more severe diseases with multiorgan involvement. This difference is important and should be remembered when dealing with patients with suspected TB infection, since the treatment and clinical course may be vastly different when other systems are involved.^3,16,17^

The diagnosis of UGTB has been reported as difficult to obtain^3,13^ and should include a combination of at least urine sediment analysis, smear and culture for acid-fast bacilli, and PCR for *MTc* in urine, with histology being obtained only when adequate or needed. Overall, in our series, the proportions of positive tests were similar to previous studies, with no single test (among those who allow TB diagnosis) having a sensitivity > 85%. These findings consolidate the need for performing multiple tests when there is suspicion of *MTc* infection.^5,16,18,19^

Antibiotic resistance was low, with only 7 (5.6%) of patients having strains resistant to at least 2 of the classical antibiotics, in line with the Japanese experience reported by Nakane et al.^13^ It is important to monitor the prevalence of multiresistant *MTc* as it can be an emerging problem. The prevalence of multidrug-resistant strains, most commonly through resistance to isoniazid and rifampicin, should be monitored as a public health measure.^20^

As previously described, the need for surgery was more common for patients with only UGTB, with extirpative surgery being performed when patients were rendered with a non-functioning organ due to chronic fibrosis leading to persistent symptoms. The kidney was the most commonly affected organ in our series, which is consistent with previous studies, which also report a low but diverse number of operations.^19,21,22^

If we think of UGTB and DTU with urinary involvement as part of a spectrum of a disease, it would be expected that those with systemic disease had a higher mortality rate. That happened in our series, with the mortality rate 3 months after diagnosis being 8.8% for patients with UGTB and 21.3% for patients with DTU, which is in line with other papers.^8,9,13^ National studies from Turkey^19^ and Japan^13^ reported low mortality rates for UGTB, both close to 1.3%. However, in DTB, studies categorically point toward a worse outcome, with studies such as those from Wang et al^8^ or Meira et al^9^ reporting mortality rates of 31.1% and 21%, respectively, most of them resulting from TB infection or overinfection.

Diagnosis is often delayed, either for late presentation or late identification of the problem. Interestingly, a Russian study reported 25.8% incidence of UGTB in patients diagnosed previously with recurrent UTI for more than 2 years^17^. As TB of the urogenital tract is a treatable disease, its early diagnosis is important in order to prevent complications including renal insufficiency, bladder fibrosis, and death. Clinicians practicing in regions with a higher incidence of the disease should bear in mind its different presentations and the modalities of diagnosis at their disposal. As Kulchavenya et al^10^ stated, we must keep in mind this diagnosis, which remains an enigma, and sometimes more art than science.

Our data consist of a population mainly with multisystem disease (82%), male patients (76%), with ages > 60 years (67%), with kidney TB (92%), with no need for urological surgery (84%), and with an important death rate in 3 months after diagnosis (19%). While data on patient gender and age are comparable on most series, most studies only include patients with TB affecting only the urogenital system, and as such report lower mortality and mostly lower urinary tract symptoms, hematuria, or lumbar pain.^11,13,19,23^ In our study, the rate of surgery was higher than most studies on the UGTB patients (71 vs. 40-50%) but lower if including all patients (5 vs. 40-50%), and general symptoms were predominant due to a predominance of DTU patients.

There are some limitations in our study: retrospective design; data from only one center, lack of information about medical treatment and treatment outcomes, and lack of standardized diagnostic approach with different tests applied to different patients.

Clinicians should remember *MTc* infection as a possible diagnosis in atypical presentations or persistent symptoms after adequate treatment. In the presence of patients with systemic symptoms, particularly risk factors such as being injected drug users or immunosuppression are present, we should be aware of the possibility of disseminated TB and remember that these patients need surgery less frequently but entail a worst disease outcome.

## Figures and Tables

**Supplementary Table 1.** Patient Data Based on Affected Organs

Features, n/N (%)	Kidney TB grade 1	Kidney TB grade 2	Kidney TB grade 3	Kidney TB grade 4	Disseminated TB (DTU)	Testicular/Epididymal TB	Prostate TB	UGTB + DTU (All cases)
N	132	6	6	15	141	4	3	172
0-20 years	1 (1)	0 (0)	1 (17)	0 (0)	0 (0)	0 (0)	0 (0)	2 (1)
21-40 years	48 (36)	1 (17)	1 (17)	1 (7)	50 (35)	1 (25)	1 (33)	54 (31)
41-60 years	56 (42)	4 (67)	3 (50)	5 (33)	62 (44)	0 (0)	1 (33)	69 (40)
>60 years	27 (20)	1 (17)	1 (17)	9 (60)	29 (21)	3 (75)	1 (33)	47 (27)
Male gender	108 (82)	1 (17)	4 (67)	8 (53)	113 (80)	4 (100)	3 (100)	130 (76)
Malaise	113 (86)	3 (50)	2 (33)	6 (40)	125 (89)	0 (0)	2 (67)	129 (75)
Anorexia	75 (57)	1 (17)	1 (17)	3 (20)	80 (57)	0 (0)	0 (0)	81 (47)
Fever	81 (61)	3 (50)	0 (0)	6 (40)	88 (62)	0 (0)	1 (33)	91 (53)
Dysuria	12 (9)	4 (67)	2 (33)	7 (47)	15 (11)	1 (25)	1 (33)	30 (17)
Storage symptoms	13 (10)	2 (33)	3 (50)	8 (53)	13 (9)	1 (25)	0 (0)	29 (17)
Hematuria	2 (2)	0 (0)	4 (67)	2 (13)	4 (3)	0 (0)	0 (0)	10 (6)
Scrotal mass	0 (0)	0 (0)	1 (17)	1 (7)	0 (0)	2 (50)	0 (0)	4 (2)
Sterile pyuria	104/132 (79)	4/6 (67)	6/6 (100)	14/15 (93)	110/141 (78)	1/4 (25)	3/3 (100)	138 (80)
*MTc* in urine	107/132 (81)	5/6 (83)	4/5 (80)	11/14 (79)	115/141 (82)	0/0 (0)	2/3 (67)	134 (78)
PCR in urine	91/117 (78)	4/5 (80)	5/5 (100)	9/11 (82)	100/127 (79)	1/1 (100)	2/3 (67)	116 (67)
Ultrasound +	5/122	6/6	5/5	14/15	29/130	4/4	1/3	35/155
CT scan +	7/28	6/6	6/6	15/15	22/38	N/A	2/3	36/58
Ureteral TB	0 (0)	4 (67)	3 (50)	3 (20)	5 (4)	0 (0)	0 (0)	14 (8)
Bladder TB	0 (0)	1 (17)	1 (17)	2 (13)	2 (1)	0 (0)	0 (0)	7 (4)
DTU	125 (95)	3 (50)	1 (17)	7 (47)	141 (100)	0 (0)	2 (67)	141 (82)
Bilateral disease	0 (0)	1 (17)	0 (0)	1 (7)	0 (0)	0 (0)	0 (0)	3 (2)
Need uro surgery	2 (2)	3 (50)	5 (83)	11 (73)	7 (5)	4 (100)	0 (0)	28 (16)
3 month death from TB	29 (22)	0 (0)	1 (17)	0 (0)	30	0 (0)	0 (0)	33 (19)

**Table 1.** Epidemiologic and Clinical Data

		Urogenital Tuberculosis	Disseminated Tuberculosis	*P*
N		31	141	
Male gender, N (%)		17 (55)	113 (80)	.005
Age, median (P25-P75)		64 (48-73)	45 (38-56)	.001
History	All risk factors, N (%)	7 (23)	118 (84)	.005
	Injected drugs users, N (%)	1 (3)	67 (48)	
	HIV infection, N (%)	1 (3)	89 (63)	
	Active chemotherapy, N (%)	2 (6)	0 (0)	
	Other medication, N (%)	4 (13)	12 (9)	
Symptoms	Malaise	4 (13)	125 (89)	<.001
	Anorexia	1 (3)	80 (57)	<.001
	Fever	3 (10)	88 (62)	<.001
	Dysuria	15 (48)	15 (11)	<.001
	Storage symptoms	16 (52)	13 (9)	<.001
	Hematuria	6 (19)	4 (3)	.003
	Scrotal mass	4 (13)	0 (0)	<.001
Diagnosis^σ^	Sterile pyuria, N (%)	28 (90)	110 (78)	.141
	*MTc* in urine, N (%)	19 (61)	115 (82)	.582
	Positive urine PCR, N (%)	16 (52)	100 (79)	.765
Affected organs	Kidney, N (%)	23 (74)	136 (96)	<.001
	Ureter, N (%)	9 (29)	5 (4)	<.001
	Bladder, N (%)	5 (16)	2 (1)	.003
	Testicle/epididymis, N (%)	4 (13)	0 (0)	<.001
	Prostate, N (%)	1 (3)	2 (1)	.451
Need urological surgery, N (%)		22 (71)^*^	7 (5)^¥^	<.001
Death 3 months after diagnosis related to* MTc* infection, N (%)		3 (10)	30 (21)	<.001

*Mtc, Mycobacterium tuberculosis complex*; PCR, polymerase chain reaction.

^*^Two ureteral stenting, 12 nephrectomies, 3 cystectomies, 1 TUR-P, 7 orchiectomies, 2 TUR-B, 1 ureteroscopy, and 1 bladder augmentation.

^¥^Two ureteral stenting, 2 nephrectomies, 3 cystectomies, 1 exploration laparoscopy, 1 ureteral reimplantation, and 1 ureteroscopy.

^σ^Percentages of diagnostic tests refer only to those who took them.

**Table 2.** Antibiotic Resistance Among Patients with Positive Mycobacterial Cultures

		Urogenital Tuberculosis (n = 18)	Disseminated Tuberculosis (n = 108)	Total (n = 126)
Isoniazid resistance		4 (22.2)	6 (5.6)	10 (7.9)
Streptomycin resistance		4 (22.2)	10 (9.3)	14 (11.1)
Ethambutol resistance		0 (0)	1 (0.9)	1 (0.8)
Rifampicin resistance		1 (5.6)	3 (2.8)	4 (3.2)
≥2 Antibiotic resistances		3 (16.7)	4 (3.7)	7 (5.6)
≥3 Antibiotic resistances		1 (5.6)	1 (0.9)	2 (1.6)
≥4 Antibiotic resistances		0 (0)	1 (0.9)	1 (0.8)
